# Genotyping of *Mycobacterium tuberculosis* complex isolated from humans and animals in northeastern Iran

**DOI:** 10.1038/s41598-023-33740-9

**Published:** 2023-04-25

**Authors:** Kiarash Ghazvini, Reza Khoshbakht, Keyvan Tadayon, Nader Mosavari, Hamid Reza BahramiTaghanaki, Gholam Reza Mohammadi, Mohammad Rashti Baf, Kimiya Nourian, Amin Samiei, Mahdis Ghavidel

**Affiliations:** 1 Antimicrobial Resistance Research Center, Mashhad University of Medical Sciences, Mashhad, Iran; 2 Department of Microbiology and Virology, School of Medicine, Mashhad University of Medical Sciences, Mashhad, Iran; 3 Department of Laboratory Sciences, School of Paramedical Sciences, Mashhad University of Medical Sciences, Mashhad, Iran; 4 Student Research Committee, Mashhad University of Medical Sciences, Mashhad, Iran; 5 Department of Microbiology, Razi Vaccine and Serum Research Institute (RVSRI), Agricultural Research, Education and Extension Organization (AREEO), Karaj, Iran; 6 PPD Tuberculin Department, Razi Vaccine and Serum Research Institute, (RVSRI), Agricultural Research, Education and Extension Organization (AREEO), Karaj, Iran; 7 School of Persian Medicine, Mashhad University of Medical Sciences, Mashhad, Iran; 8 Department of Clinical Sciences, School of Veterinary Medicine, Ferdowsi University of Mashhad, Mashhad, Iran; 9 Deputy of Veterinary Administration of Khorasan Razavi Province, Mashhad, Iran; 10 Department of Veterinary Medicine, Ferdowsi University of Mashhad, Mashhad, Iran; 11 Tuberculosis and Leprosy Coordinator at Health Chancellor, Health Center of Khorasan State, Mashhad University of Medical Sciences, Mashhad, Iran; 12 Shahid Hasheminejad Hospital, Mashhad University of Medical Sciences, Mashhad, Iran

**Keywords:** Bacteriology, Medical research, Molecular medicine

## Abstract

The objective of this study was to genotype *Mycobacterium tuberculosis* complex isolated from humans and cattle in northern Iran. Over the course of one year, a total of 120 human and 21 cattle isolates were tested using region of difference (RD)-based polymerase chain reaction (PCR) and mycobacterial interspersed repetitive unites-variable number tandem repeats (MIRU-VNTR). In *M. tuberculosis,* out of 120 isolates investigated, the most common genotype detected was NEW-1 (53.3%), followed by CAS/ Delhi (24.1%), Haarlem (5%), Beijing (4.16%), Uganda I (4.16%), S (3.3%), Ural (0.83%), TUR (0.83%), Uganda II (0.83%), Lam (0.83%) and Cameroon (0.83%). The HGDI rate was 0.9981 and the clustering rate was 10.83. Of the isolates, QUB26 had the highest allele diversity (h: 0.76), while the loci Mtub29 and MIRU24 had the lowest (h: 0). In *M. Bovis*, out of 123 collected tissue samples, 21 (17%) grew on culture media. The HGDI rate was 0.71 and clustering rate was 85.7%. The locus ETRC had the highest allele diversity (h: 0.45). The findings of this study suggest that there is high genetic diversity among *M. tuberculosis* isolates in Khorasan Razavi Province, which is consistent with similar results from other studies in other provinces in Iran and neighboring countries. This indicates that the prevalent genotypes in this study are spreading in the Middle East region. Furthermore, considering that *M. Bovis* isolates were identified in two clusters, it seems that all of them have a common origin and are circulating among the livestock farms in the province.

## Introduction

*Mycobacterium tuberculosis* complex is responsible for causing tuberculosis (TB). Before the onset of Covid-19, TB was the leading cause of death, even more than HIV/acquired immune deficiency syndrome (AIDS). The lungs are the main site of infection; however, the infection can spread to other parts of the body. It is estimated that two billion people, that is a quarter of the world’s population, have been infected with TB^1,2^ In 2021, more than 10 million new cases of TB appeared, with an 4.5% increase from 2020. Also, WHO reported 1.6 million people died from TB in 2021^3^. *Mycobacterium tuberculosis* (*M. tuberculosis*) and *Mycobacterium bovis* (*M. bovis*) are two significant contributors of the *Mycobacterium tuberculosis* complex. *M. tuberculosis* is the primary cause of TB in humans and is transmittable through aerosols an infected person coughs, sneezes, spits or even talks^4,5^. *M. bovis* is responsible for bovine tuberculosis (bTB) and has the widest range of hosts among *Mycobacterium tuberculosis* complex. Cattle is its main host, and it can be spread to other hosts through the respiratory route; however, ingestion of infected food and water can also lead to infection^6^. *M. bovis* is also capable of causing TB in humans, which is known as a neglected zoonotic disease. If humans consume unpasteurized dairy products, undercooked meat, or come into direct contact with an infected animal, they can fall prey to this disease^7,8^. Thus, it is essential to detect and diagnose it in human populations. To prevent outbreaks of *M. bovis* in human populations, tests and slaughter policies have been adopted. This means that if the comparative intradermal tuberculin test (single intradermal comparative cervical tuberculin test, SICC) of cattle comes back positive, then the animal is considered a reactor and must be sent to the slaughterhouse^9^. Epidemiological studies are regarded as being extremely significant in many aspects, including disease management, detecting the source of infection, identifying transmission routes, understanding the relationships between different outbreaks and tracking specific strains of pathogens to understand the distribution of disease in populations^9,10^. In order to follow the progression of TB, it is essential to compare the profiles of various isolates using advanced molecular-based techniques such as mycobacterial interspersed repetitive unites-variable number tandem repeats (MIRU-VNTR) analysis, Spacer Oligonucleotide typing (Spoligotyping), Restriction Fragment Length Polymorphism (RFLP) analysis and Next Generation Sequencing (NGS) in strain typing^11–14^. The aim of this study was to investigate, for the first time in Iran, the genetic diversity of *M. tuberculosis* and *M. bovis* using 24 loci MIRU-VNTR in Khorasan Razavi Province. Furthermore, this study also sought to investigate the possibility of isolating *M. bovis* from humans and *M. tuberculosis* from cattle.

## Results

### Region of difference (RD)-based PCR

Among the 120 human samples tested by RD typing, all isolates were identified as *M. tuberculosis.* Of the 123 cultured cattle tissue samples, 21 strains grew on pyruvate Lowenstein–Jensen media, which were identified as *M. bovis* in RD typing.

### Demographic data of patients used for *M. tuberculosis* isolation

120 *Mycobacterium tuberculosis* isolates were collected from 63 male (52.5%) and 57 female (47.5%) patients. Five of the patients were from Afghanistan, while the remaining 115 had Iranian nationality. Out of these, five had prior prison records. The age of the patients varied between 19 and 97, with an average age of 60; 14 (11.6%) patients were aged 19–30, 17 (14.1%) aged between 31 and 45; 26 (21.6) aged 45–65, and 63 (52.5%) were older than 65.

### Mycobacterial interspersed repetitive unites-variable number tandem repeat (MIRU-VNTR) analysis of human isolates

Out of the 120 investigated isolates, 95 (79.16%) had patterns similar to those in the MIRU-VNTR plus database. These included genotypes such as NEW-1 (59 isolates, 49.17%), CAS/Delhi (21 isolates, 17.5%), Beijing (5 isolates, 4.17%), S (3 isolates, 2.5%), Uganda I (2 isolates, 1.67%), Ural (1 isolate, 0.83%), TUR (1 isolate, 0.83%), Uganda II (1 isolate, 0.83%), Lam (1 isolate, 0.83%) and Cameroon (1 isolate, 0.83%). The remaining 25 isolates showed no similar patterns to those of the MIRU-VNTR plus database, so they were considered as unknown patterns. To identify these genotypes, we compared the 25 isolates to the patterns of TB miner database, resulting in the genotypes of 23 isolates being identified, including CAS/Delhi (8 isolates), NEW-1 (5 isolates), Haarlem (6 isolates), Uganda I (3 isolates) and S (1 isolate).

**Figure 1:**
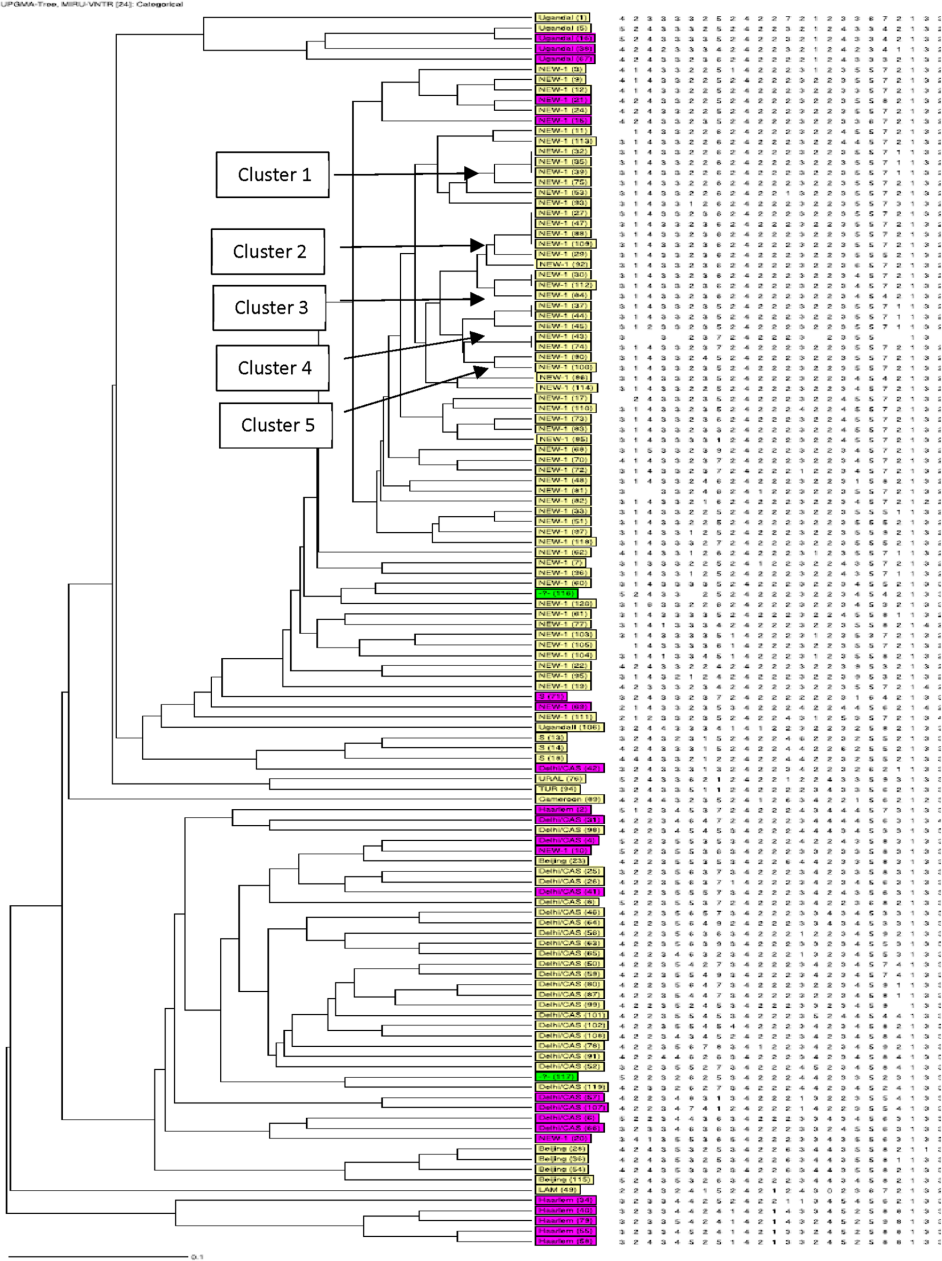
UPGMA tree showing genetic relatedness of 120 isolates from Khorasan Razavi province based on the 24-locus MIRU-VNTR. Genotype and copy numbers are mentioned for each isolate along with the dendrogram, except for isolate with polyclonal infection. The arrangement of MIRU-VNTR loci from left to right including: MIRU 02, Mtub04, ETR C, MIRU 04, MIRU 40, MIRU 10, MIRU 16, Mtub21, MIRU 20, QUB-11b, ETR A, Mtub29, Mtub30, ETR B, MIRU 23, MIRU 24, MIRU 26, MIRU 27, Mtub34, MIRU 31, Mtub39, QUB-26, QUB-4156, MIRU 39. Yellow color genotypes were identified by MIRU-VNTR plus, Violent color genotypes were identified by TB miner and green color genotypes were unknown.

The 120 M*. tuberculosis* isolates exhibited 112 different patterns. Thirteen of these isolates were categorized in five clusters; all of which belonged to NEW-1 genotype. Cluster one comprised three genotypes, which cluster four encompassed four genotypes. The remaining clusters each included two genotypes of NEW-1 (Fig. 1).

The three isolates had been categorized in cluster one, one of which had been collected from an Afghan patient, another from an individual with a prison record, and the last from a female patient. All five patients of Afghan nationality had diverse genotypes, including NEW-1, CAS/Delhi, Beijing, Haarlem, and Uganda I. This suggests diversity among Afghan people.

**Figure 2:**
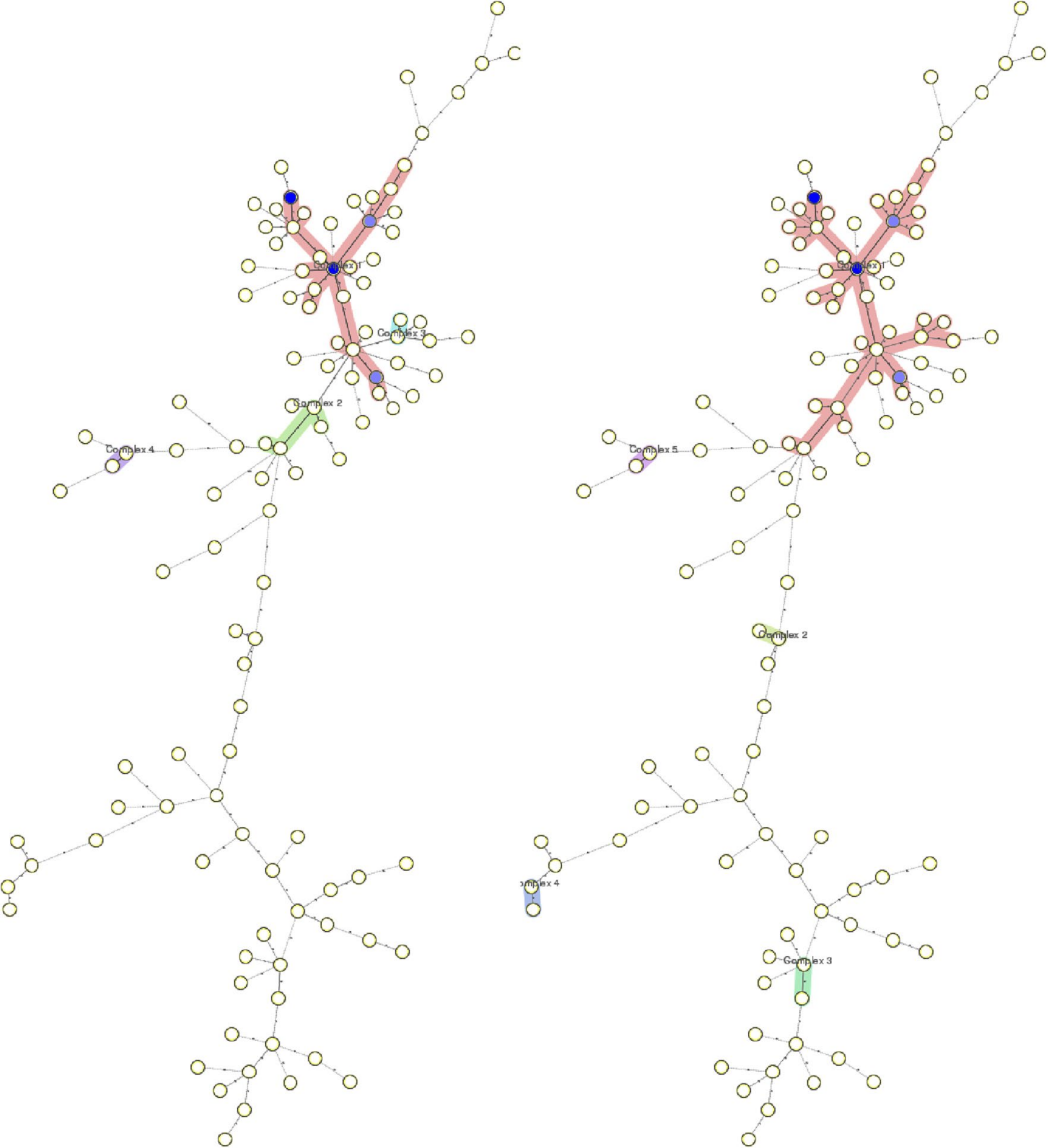
Minimum spanning tree (MST) based on 24-locus MIRU-VNTR demonstrating lineage classification of the 120 Mycobacterium tuberculosis isolates. (**A**): MST based on SLV (Single Locus Variation), (**B**): MST based on DLV (Double Locus Variation).

In order to better identify the clonal complex among the isolates, we drew Minimum Spanning Trees (MST) based on Single Locus Variants (SLV) and Double Locus Variants (DLV). We identified 31 isolates classified into 4 clonal complexes when drawing MST based on SLV, and 50 isolates classified into 5 clonal complexes when drawing MST based on DLV, (as shown in Fig. 2). Based on SLV, *M. tuberculosis* isolates were identified in four clonal complexes, the first three clonal complexes (CC1,CC2, CC3) belonged to the NEW-1 genotype and each had 23, 4 and 2 isolates, respectively. The fourth clonal complex (CC4) belonged to Uganda I genotype and had 2 isolates. Based on DLV, *M. tuberculosis* isolates were identified in five complexes. First clonal complex (CC1) belonged to the NEW-1 genotype with 42 isolated. Genotype of the second clonal complex (CC2) was Beijing, the third (CC3) was CAS/Delhi, the fourth (CC4) was Haarlem, and the fifth (CC5) was Uganda I, which all of the complexes had 2 isolates.

Seven isolates were identified that had two or three bands in one or more loci. Of these isolates, five had two alleles in one locus, while two others had the same number of alleles in more than two loci. One of these isolates possessed two bands at loci ETR B, ETR E, ETR C, QUB4156, QUB26, Mtub04 and Mtub34, and was isolated from a patient who passed away within a month. Another isolate also had two alleles in the loci ETR B and ETR C. The remaining five isolates were found to have two alleles in the loci QUB4156, MIRU10 and ETR A.

### Allelic diversity of *M. tuberculosis*, discriminatory power and recent transmission

The high genotyping discrimination index (HGDI) for MIRU-VNTR typing was 0.9981 and the clustering rate was 10.83%. Furthermore, the rate of TB recent transmission was 6.66%. The allelic diversity of the MIRU-VNTR loci was estimated, with 6 loci showing high diversity, 10 loci showing moderate diversity and the rest of the loci showing low allele diversity. The locus QUB26 had the highest allelic diversity (h = 0.76), while the loci Mtub29 and MIRU24 had the lowest allelic diversity (h = 0). Table 1 displays the number of alleles and estimated allele diversity for the loci, as well as their classification.

**Table 1:** Allelic diversity for each locus for *Mycobacterium tuberculosis* isolates.

Locus	Allele number										h	Conclusion
	1	2	3	4	5	6	7	8	9	10		
QUB26	–	3	7	8	12	10	49	23	–	7	0.76	High
MIRU26	5	3	2	9	46	32	18	1	4	–	0.75	High
MIRU10	5	54	21	7	15	15	1	1	–	–	0.72	High
Mtub21	3	10	21	37	45	1	–	–	2	–	0.72	High
MIRU16	7	41	54	15	2	–	1	–	–	–	0.66	High
QUB4156	16	71	19	9	–	–	–	4	–	–	0.62	High
ETRA	–	3	61	42	11	–	–	–	–	–	0.59	Moderate
Mtub04	10	71	15	21	–	–	–	2	–	–	0.59	Moderate
ETRE	–	5	74	14	26	–	–	–	–	–	0.55	Moderate
ETRC	–	33	9	74	1	1	–	–	–	–	0.52	Moderate
ETRB	55	61	2	–	–	–	–	–	–	–	0.51	Moderate
Mtub34	–	63	54	2	–	–	–	–	–	–	0.51	Moderate
MIRU39	10	79	29	1	1	–	–	–	–	–	0.5	Moderate
Mtub39	–	5	87	21	6	1	–	–	–	–	0.44	Moderate
MIRU40	6	8	92	13	–	–	1	–	–	–	0.39	Moderate
QUB11b	3	99	4	8	–	5	1	–	–	–	0.31	Moderate
Mtub30	1	101	–	18	–	–	–	–	–	–	0.27	Low
MIRU23	–	–	5	–	109	6	–	–	–	–	0.17	Low
ETRD	2	–	115	3	–	–	–	–	–	–	0.08	Low
MIRU27	1	2	114	3	–	–	–	–	–	–	0.09	Low
MIRU20	5	115	–	–	–	–	–	–	–	–	0.08	Low
MIRU2	5	115	–	–	–	–	–	–	–	–	0.08	Low
Mtub29	–	–	–	120	–	–	–	–	–	–	0	Low
MIRU24	120	–	–	–	–	–	–	–	–	–	0	Low

### Demographic data of cattle used for the isolation of *M. bovis*

In this study, 123 samples were collected from cattle with positive tuberculin test from 30 cattle farms that had been sent to Mashhad industrial slaughterhouse from May 2017 to May 2018. The results from the cultured samples revealed that 21 (17%) of these samples tested positive for *M. bovis*. The studied livestock population comprised of 119 cows (96.7%) and 4 bulls (3.25%). Among them, 83 cows were not pregnant, while 16 were pregnant with fetuses less than 5 months old. Of the pregnant cows, 8 had fetuses estimated to be between five and seven months old, and 12 cows had passed their seventh month of gestation. The youngest animal was a three-month-old calf, and the oldest was an 81 month-old-cattle. The lowest weight for these cattle was 60 kg, and the highest was 850 kg. Of the samples collected from the cattle, 66 (53.7%) had no granulomatous lesions (Non visible lesion = NVL) while 57 (46.3%) showed infected organs (visible lesion = VL).

### MIRU-VNTR analysis of cattle isolates

After analyzing the 21 M*. bovis* isolates from 5 cattle farms in Khorasan Razavi (3 farms in Mashhad, 1 in Chenaran and 1 in Gonabad) in a database, it was identified that they were *M. bovis*.

**Figure 3:**
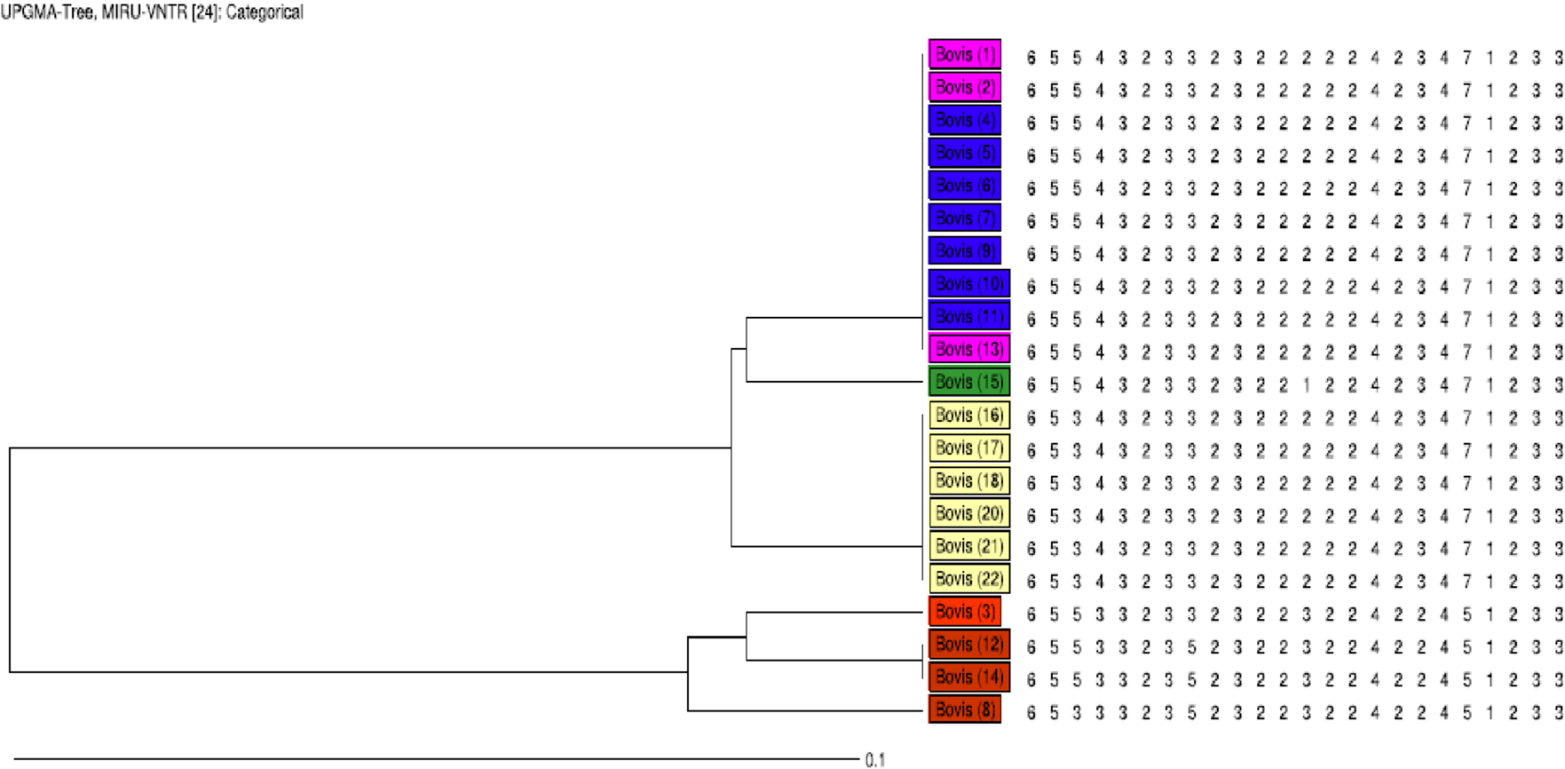
UPGMA tree showing genetic relatedness of 21 *Mycobacterium bovis* isolates from Khorasan Razavi province based on the 24-locus MIRU-VNTR. Genotype and copy numbers are mentioned for each isolate along with the dendrogram The arrangement of MIRU-VNTR loci from left to right including: MIRU 02, Mtub04, ETR C, MIRU 04, MIRU 40, MIRU 10, MIRU 16, Mtub21, MIRU 20, QUB-11b, ETR A, Mtub29, Mtub30, ETR B, MIRU 23, MIRU 24, MIRU 26, MIRU 27, Mtub34, MIRU 31, Mtub39, QUB-26, QUB-4156, MIRU 39. Violent color strains were isolated from farm1 (Mashhad), blue color strains were isolated from farm 2 (Mashhad), green color strain was isolated from farm 3 (Gonabad), yellow color strains were isolated from farm 4 (Mashhad) and red color strains were isolated from farm 5 (Chenaran).

These 21 isolates exhibited 6 distinct patterns and 18 of them were divided into 3 clusters. Cluster 1 consisted of 10 isolates, 3 of which originated from a cattle farm in Mashhad and the rest (7) from a farm located in another region of the city. Cluster 2 included 6 isolates, all collected from a single farm in Mashhad. Lastly, Cluster 3 had 2 isolates, collected from a farm in Chenaran (Fig. 3).

**Figure 4:**
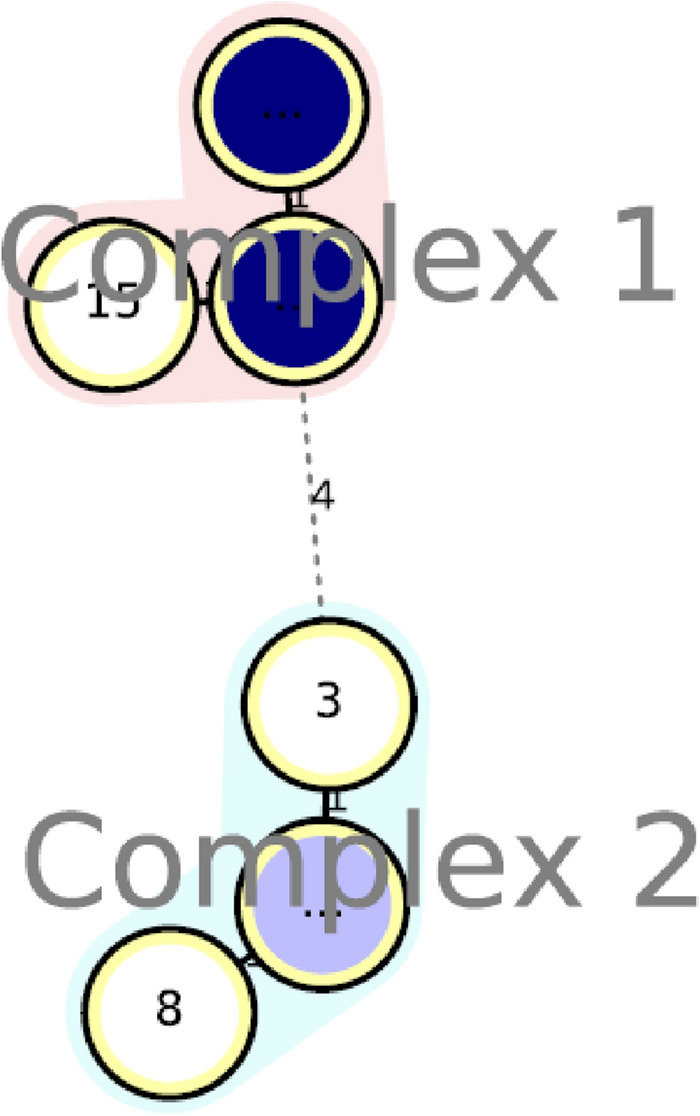
Minimum spanning tree (MST) based on 24-locus MIRU-VNTR demonstrating lineage classification of the 21 *Mycobacterium bovis* isolates. MST based on SLV (Single Locus Variation).

Drawing MST was based on SLV suggesting that twenty-one isolates belonged to two clonal complexes: CC1 had 17 isolates and CC2 had 4 isolates (Fig. 4).

### Allelic diversity, discriminatory power and recent transmission

The high genotyping discrimination index for MIRU-VNTR typing was 0.71, and the rate of clustering was 85.7%. The rate of bTB recent transmission was estimated to be 71.4% in the livestock population of Khorasan Razavi. The allelic diversity of all MIRU-VNTR loci was estimated among the investigated loci, 2 loci showed moderate allelic diversity, while the diversity of the other loci was low. The locus ETRC had the highest allele diversity (h = 0.45). The table shows the number of alleles and estimated allele diversity (Table 2).

**Table 2:** Allelic diversity for each locus *for Mycobacterium bovis* isolates.

Locus	Allele number							h	Conclusion
	1	2	3	4	5	6	7		
ETRC	–	–	7	–	14	–	–	0.45	Moderate
QUB11b	1	16	3	–	–	–	–	0.38	Moderate
QUB26	–	–	–	–	4	–	17	0.27	Low
Mtub21	–	4	17	–	–	–	–	0.27	Low
ETRD	–	–	4	17	–	–	–	0.27	Low
MIRU26	–	–	18	–	3	–	–	0.24	Low
MIRU16	–	–	21	–	–	–	–	0	Low
QUB4156	21	–	–	–	–	–	–	0	Low
ETRA	–	–	–	–	–	21	–	0	Low
Mtub04	–	21	–	–	–	–	–	0	Low
ETRE	–	–	21	–	–	–	–	0	Low
MIRU10	–	21	–	–	–	–	–	0	Low
ETRB	–	––	–	–	21	–	–	0	Low
Mtub34	–	–	21	–	–	–	–	0	Low
MIRU39	–	21	–	–	–	–	–	0	Low
Mtub39	–	21	–	–	–	–	–	0	Low
MIRU40	–	21	–	–	–	–	–	0	Low
Mtub30	–	–	–	21	–	–	–	0	Low
MIRU23	–	–	–	21	–	–	–	0	Low
MIRU27	–	–	21	–	–	–	–	0	Low
MIRU20	–	21	–	–	–	–	–	0	Low
MIRU2	–	21	–	–	–	–	–	0	Low
Mtub29	–		21	–	–	–	–	0	Low
MIRU24	–	21	–	–	–	–	–	0	Low

### Transmission of *M. bovis* from cattle to humans

Among the twenty-seven farm workers, two people had a suspicious PPD test, with the diameter of the skin induration measuring 8 and 9 mm. To verify the infection, septum samples were collected, cultured, and then PCR was performed using specific primers for *Mycobacterium* genus. Chest X-rays was performed for these two people, however, no pathologic signs were found.

## Discussion

In the present study, 120 M*. tuberculosis* isolates were investigated using MIRU-VNTR. We found 112 different patterns and 13 isolates were categorized into 5 clusters. The Most prevalent genotype was NEW-1, accounting for 53.3%. Previous studies in Iran reported the prevalence of NEW-1 genotype to be 32.3% in Golestan, 22.5% in Tehran and 0.65% in East Azerbaijan provinces. The high prevalence of this dominant genotype in Golestan and Tehran are consistent with our results suggesting that Iran has been a transmission route for this genotype for a long time. Furthermore, studies carried out in neighboring countries showed the abundance of NEW-1 genotype in the common borders of Turkmenistan and Uzbekistan to be 4.6% and 1.9%, respectively, and in southern regions of Kazakhstan^17–22^.

Based on the present results, CAS/Delhi was the second most abundant genotype. It was isolated in various parts of Iran, including Khorasan Razavi, Golestan, Hormozgan and Tehran. The prevalence of this genotype varied from 1.25 to 40.8%. Iran’s neighboring such as Saudi Arabia, India, Pakistan and Afghanistan have all reported a high prevalence of CAS/Delhi genotype^17,18,23–30^. This indicates that the genotype is widely spread across the region, possibly due to migration and inter-country movement.

Haarlem genotype had been previously identified as the third most common genotype in many regions of Iran, such as Khorasan Razavi, Hormozgan, Khuzestan, East Azerbaijan and Tehran. Its abundance ranged from 0.65% in Tabriz to 47.9% in Khorasan. Additionally, two studies conducted in Brazil and Colombia showed that the prevalence of this genotype was 21.5% and 38.25%, respectively^17,19,23,24,31–33^. Evidently, Haarlem genotype was a widespread strain in the Middle East and Latin America.

There have been three previous studies regarding the circulation of Beijing genotype in Iran, ranging from 1.7 to 24%. Additionally, this genotype has been located in the northern neighboring countries of Iran, including Kazakhstan, Georgia, Russia, as well as countries in Central and East Asia^34–41^. This indicates that the Beijing genotype is spreading across the region, and highlights the importance of further research into its circulation and how it can be better managed.

These differences in identifying genotypes across different regions can be attributed to a number of factors, such as the number of loci used, the number of cases studied, the duration of the study, and most importantly, the geographical region. After all, it stands to reason that the geography of an area will play a significant role in the genotypes found. Studies conducted in East Azarbaijan and Golestan provinces, which used 12, 15 and 24 MIRU-VNTR as their typing methods, reported the rate of recent transmission to be between 19 and 27%. Additionally, related studies from China, London, and Netherlands indicated a rate of recent transmission ranging from 13.5 to 35%^17–19,42–45^. In contrast to these studies, results from MIRU-VNTR typing in this study showed that tuberculosis transmission from recent infection was responsible only for 6.66% of cases. Based on high diversity of isolates and the low transmission rate, it has been suggested that reactivation of latent infection has play a central role in the incidence of the disease in this region. This is supported by the fact that 52.5% of our patients were aged over 65. According to the recently revised guidelines from the National Institute for Health and Care Excellence (NICE), it is advised that people between the ages of 18 to 65 should be screened, as latent tuberculosis can reactivate after the age of 65^46^. Various factors can cause tuberculosis bacteria to transform from its latent form to its reactivated form, such as age, race, occupation, economic situation, hygienic gear, immigration, addiction, underlying disorders like diabetes, cancer, and respiratory failure^47^.

Analysis of the results of MIRU-VNTR typing revealed that 10.83% of the identified isolates were categorized into clusters. In other studies conducted in Iran, the clustering ratio varied between 0.0 and 75%. The major reason for such discrepancies is the number of loci used; as the number of loci increases, the clustering ratio decreases due to the increased discriminatory power^17,19,34,35,48,49^. In our study, we established a high discriminatory power for MIRU-VNTR typing of 0.998 (HGDI). This was higher than the rate reported in previous studies in Iran, which ranged from 0.972 to 0.993. In investigations conducted in other countries, this rate has been reported to be between 0.920 and 1.000^19,24,25,27,49–51^.

By investigating other studies, six loci were found to have the highest allelic diversity including MIRU10, MIRU26, QUB26, MIRU40, QUB11b and Mtub21. In the present study, the loci QUB26, MIRU26, MIRU10, Mtub21, MIRU16 and QUB4156 were the ones with h ˃ 0.6; both results contained four identical loci. On the other hand, the results of the other studies, which suggested ETRD and MIRU20 had the lowest allelic diversity, were in accordance with our results^17,18,32,50,52–55^. We showed that seven isolates had multiple alleles in one or more loci. Two of them had two alleles in more than one locus, suggesting polyclonal infection, while five isolates had two alleles in one locus, indicating microevolution. Previous studies in Iran have reported a polyclonal infection rate ranging from 1.6 to 26.6%, while other countries have reported this rate to be between 0.6 and 19.3%. Moreover, one isolate had three different copy number variants (CNVs) in the locus MIRU10, and in the past, three CNVs were reported in the loci MIRU24 and Mtub30. These isolates could be considered for new algorithm (e.g., Class TR) for classification of complex infections^17,36,56–62^.

This study aimed to uncover the relationship between different isolates by drawing a Minimum Spanning Tree (MST). The results revealed that Clonal Complex 1 was concentrated on the right side and related to Clonal Complexes 2 and 3. This complex was composed of 23 isolates belonging to the NEW-1 genotype. Interestingly, isolates with longer branches connected to the central region of this complex could be a sign of ancient transmission. Clonal Complex 4, however, was not related to any of the clonal complexes and belonged to the Uganda I genotype.

Several research studies were conducted in Iran and around the world aiming to isolate *M. bovis* from blood, milk and tissues of reactor cows. The rate of isolating *M. bovis* ranged from 5 to 46%. We cultured 123 samples from lymph nodes of tuberculin positive cattle and subsequently detected 21 isolates of *M. bovis*, which constituted 17% of the total samples^63–66^.

Among different studies, QUB11b and QUB32 exhibited the highest allelic diversity, while ETRD and MIRU10 had the lowest allelic diversity^67^. In the present study, QUB11b and ETRC were categorized as the loci with moderate discriminatory powers.

A study conducted in 2018 in Mozambique is the only research in the world to have investigated *M, bovis* diversity using the standard 24 MIRU-VNTR. Out of 59 isolates, 47 of them were categorized into nine clusters, with a HGDI of 0.87 with a clustering rate of 79.66%. Furthermore, tuberculosis caused by recent transmission was 71.18%. In contrast to our findings, the rate of clustering was increased to 85.7%, and the HGDI decreased to 0.71. Both studies had transmission rates of approximately 71%. In the Mozambique study, the loci Mtub30, MIRU23, Mtub34, ETRE, Mtub39, QUB4156, MIRU39, ETRD, MIRU20 and MIRU2 showed no allele diversity among the isolates, which is in agreement with our results, except for the ETRD locus which possessed two alleles in our research^68^.

The rate of tuberculosis transmission in livestock population of Khorasan Razavi is alarmingly high at 76.1%. Our study revealed that *M. bovis* is mostly transferred horizontally among cattle in a farm, indicating that the spread of the disease is due to the improper transfer of cattle without conforming to quarantine regulations in the dairy cattle industry. Careful management is essential as cattle are kept close to one another during milking or in the wintertime, leaving a chance for calves to be infected if they are fed the infected cow’s milk. When all isolates separated from different farms are categorized into one cluster and show a similar genetic pattern, there is only one source of infection and the disease may spread due to lack of quarantine regulations when transferring cattle from farm to farm.

In the present study, *M. bovis* was not isolated from the human population, which is in contrast to a study performed in Zambia. In the Zambian study, *M. bovis* was detected in sputum of a patient, and the cause of the disease was attributed to the consumption of unpasteurized milk^69^. Moreover, in our study, *M. tuberculosis* was not isolated from cattle lymph nodes, whereas, in another study conducted in Zambia, *M. tuberculosis* was separated from cattle, suggesting an epidemiological correlation between *M. tuberculosis* transmission in livestock and humans. In that study, they hypothesized that *M. tuberculosis* in livestock was due to livestock spill back^70^.

Khorasan Razavi province has common borders with Afghanistan and Turkmenistan, and so, further research is required to investigate the strains isolated in this province and to study the probability of the transfer of these isolates through migration or travel. Comparing the isolates from Khorasan Razavi with the ones separated from Afghan and Turkmen patients should take into account factors such as race, hygiene level, and climate. By doing so, we will be able to gain more insight into the potential for transfer and the impact of environmental conditions on the spread of disease.

It should be mentioned that further studies on the simultaneously genotyping of *M. tuberculosis* and *M. bovis* should be done in order to prevent outbreaks in the region. One of the limitations that should be addressed in the future studies would be the sample size. With higher sample size authors could give a good estimation on the results.

## Conclusion

Our results showed a high genomic diversity between isolates, and the most prevalent genotypes among *M. tuberculosis* isolates was NEW-1 (53.3%). The high diversity of circulating *M. tuberculosis* in the human population of Khorasan Razavi suggests the reactivation of latent infection, and spreading the infection in the region. Therefore, screening and treating these individuals with latent infection has a significant role in decreasing the prevalence of the disease in Khorasan Razavi.

When studying *M. bovis* isolated from the livestock population in Khorasan Razavi, it was found that all the isolates of *M. bovis* were in two clusters, indicating that the bacteria had been transferred among the cattle in this region from one origin. Therefore, it is recommended that the tuberculin test be conducted periodically in order to prevent the transmission of the disease among cattle in farms by eliminating the reactor cattle or quarantining the suspected ones.

## Methods

### Data and specimen collection

From May 2017 to May 2018, 120 *M. tuberculosis* clinical isolates were collected from sputum, bronchial alveolar lavage, and the gastric juice of the patients referred to two hospitals in Khorasan Razavi. A written informed consent was obtained from all the subjects who entered the study. In addition, demographic characteristics including age, gender, nationality and prison record were gathered from the Information Records of the Iranian TB registry program.

There were 123 samples collected from lymph nodes, liver, kidneys and lungs of reactor cattle slaughtered in the industrial slaughterhouse of Khorasan Razavi from May 2017 to May 2018. Further information about these cattle, including age, sex, pregnancy and infected organs was gathered from 30 cattle farms in 6 cities in Khorasan Razavi, such as Mashhad, Neyshabur, Torbatjam, Quchan, Chenaran and Gonabad. The samples were investigated for granulomatous lesions before being digested and decontaminated with 5 mL Nacetyl-L-cysteine/sodium hydroxide (5 g/L N-acetyl-Lcysteinein 3.5 M NaOH and 0.05 M sodium citrate) for 15 min. After that, the digested specimens were centrifuged and neutralized with HCL (0.1 N). Finally, the sediments were cultured on duplicate Lowenstein–Jensen media, one containing glycerol and the other pyruvate (Merck, Germany), and incubated at 37 °C. The culture tubes were were monitored for growth for at least 8 weeks^64^.In order to identify *M. bovis* in tuberculin-positive cattle, samples were collected after slaughter and examined as previously mentioned.

The *M. tuberculosis* complex were examined under biosafety level 3 (BSL3) condition. To ensure biosecurity, all samples were placed in zip bags and transported to the laboratory in a polypropylene plastic biojar. Once in the laboratory, the samples were examined under a Class II (laminar flow) biological safety cabinet. All methods used in this study were conducted under the supervision of Mashhad University of Medical Sciences and in accordance with the guidelines announced by this institution. Furthermore, ethical clearance was obtained from this university with the code of 941808.

### Farm workers

In order to increase the chances of detecting *M. bovis* in the human population, three cattle farms in three different regions of Khorasan Razavi with the highest number of tuberculin-positive cattle were chosen. These farms had sent their cattle to the Mashhad slaughterhouse. The PPD and TB tests were conducted on all 27 workers (17 men and 10 women) using three consecutive sputum specimens. The sputum samples were decontaminated using Petroff’s method and then cultured on duplicate Lowenstein–Jensen media. The culture tubes containing glycerol and other pyruvate (Merck, Germany) were incubated at 37 °C and checked for growth weekly for at least 8 weeks.

### DNA extraction

DNA samples were obtained from the suspended colonies in 200 µL of TE by heating them at 96 °C for 30 min. After heating the colonies, the suspension was centrifuged at 9750*g* for 15 min, and the supernatant containing the DNA was carefully removed and stored at − 20 °C for future use. This process ensured that the DNA samples were properly collected and preserved for further study^71^.

### Region of difference (RD)-based PCR

In this study, RD typing was used to differentiate and verify the detected isolates. To confirm the grown colonies of *M. tuberculosis* and *M. bovis*, RD PCR was performed using four genes: RD1, RD4, RD9 and RD12^72^.

### 24 loci Mycobacterial interspersed repetitive unites-variable number tandem repeat (MIRU-VNTR)

At first, the panel of primers for the flanking regions of 24 loci MIRU-VNTR were used to conduct separate PCRs. Then, gel electrophoresis was used to detect the length of the repeat units for each amplicon. *M. tuberculosis* H37Rv was used as the positive control, and deionized water was used as the negative control for each locus. To compare the sample band size, a 100 bp DNA ladder was used as the standard size. This standard marker was supplied by Pars Tous, Iran. To verify the results, in case an isolate demonstrate multiple bands at one locus, the PCR was repeated for that locus^73^.

### Analysis of Hunter-Gaston discriminatory index (HGDI) and the allelic diversity

The diversity for each locus was determined by allelic diversity (h) and the Hunter-Gaston discriminatory index (HGDI) was used to show the discriminatory power of MIRU-VNTR. The range for h and HGDI values ranged from 0.00 to 1.00. An h value of 0.00 meant that the allele was only detected in one specific locus, while an h value of 1.00 indicated that one precise allele played the main role in differentiating all the isolates. An HGDI value of 0.00 suggested that all isolates were identical ,while an HGDI value of 1.00 demonstrates that all isolates are different^74,75^.

### Phylogenic analysis

Using the MIRU-VNTR plus and TB miner servers, we categorized different isolates into clusters and determined their genotypes. We then drew a Minimum Spanning Tree (MST) to detect the phylogenic relationships among the isolates using the accessible tools from the MIRU-VNTR plus online database. The MST showed the clonal complexes (CCs) and the phylogenetic relationships between MIRU-VNTR profiles, which were differentiated based on genetic diversity. To assess the genetic agreement, we produced a dendogram using the unweighted-pair group method with arithmetic mean (UPGMA) algorithm.

### Ethics approval and consent to participate

The research was approved by the Ethics Committee of Mashhad University of Medical Sciences (code 941808).

## Supplementary Information

Supplementary Information.

## Data Availability

The data that support the findings of this study are available from MUMS but restrictions apply to the availability of these data, which were used under license for the current study, and so are not publicly available. Data are however available from the authors upon reasonable request from the corresponding author and with permission of MUMS.
