# Supplementary material for: Genotyping of Mycobacterium tuberculosis complex isolated from humans and animals in northeastern Iran
Source: Sci Rep. 2023 Apr 25;13:6746. doi: 10.1038/s41598-023-33740-9 (PMC10127167; doi:10.1038/s41598-023-33740-9)
Supplement: Supplementary file 1 — Supplementary Information. [file 41598_2023_33740_MOESM1_ESM.pdf]

Genotyping of Mycobacterium tuberculosis complex isolated from humans and animals in northeastern Iran

Kiarash Ghazvini, Reza Khoshbakht, Keyvan Tadayon, Nader Mosavari, Hamid Reza BahramiTaghanaki, Gholam Reza Mohammadi, Mohammad Rashti Baf, Kimiya Nourian, Amin Samiei, Mahdis Ghavidel*

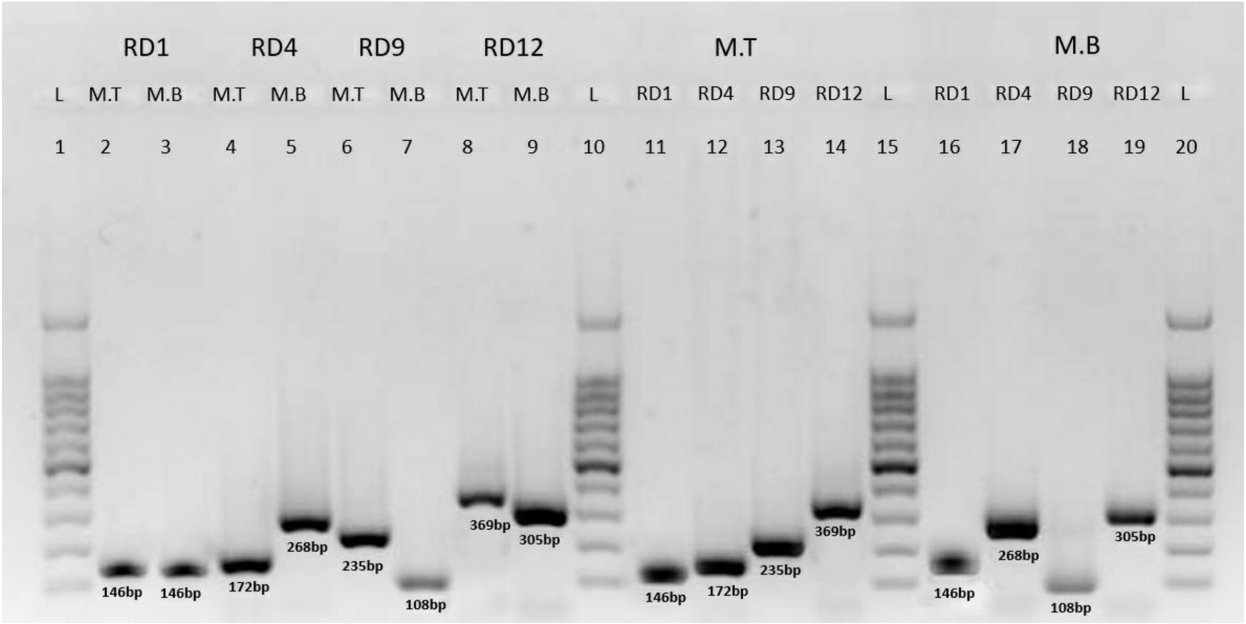

Supplementary figure 1. RD typing for *M. tuberculosis* and *M. bovis*: number 1: 100 bp ladder, number 2: RD1 for *M. tuberculosis*, number 3: RD1 for *M. bovis*, number 4: RD4 for *M. tuberculosis*, number 5: RD4 for *M. bovis*, number 6: RD9 for *M. tuberculosis*, number 7: RD9 for *M. bovis*, number 8: RD12 for *M. tuberculosis*, number 9: RD12 for *M. bovis*, number 10: 100 bp ladder, number 11: RD1 for *M. tuberculosis*, number 12: RD4 for *M. tuberculosis*, number 13: RD9 for *M. tuberculosis*, number 14: RD12 for *M. tuberculosis*, number 15: 100 bp ladder, number 16: RD1 for *M. bovis*, number 17: RD4 for *M. bovis*, number 18: RD9 for *M. bovis*, number 19: RD12 for *M. bovis*, number 20: 100 bp ladder
